# Our New York Letter

**Published:** 1887-07

**Authors:** 


### Correspondence.

### OUR NEW YORK LETTER.

INTUBATION OF THE LARYNX—HAY FEVER—THE GALVANIC CURRENT IN THE TREATMENT OF ATROPHIC RHINITIS—THE POOR CHILDREN OF NEW YORK AND HOW THEY ARE CARED FOR—A TRAINING SCHOOL FOR MALE NURSES, ETC.

*Editors Atlanta Medical and Surgical Journal:*

The American Laryngological Association held its ninth annual congress in the Academy of Medicine building. After the President, Dr. E. F. Ingals from Chicago, had delivered the opening address he read a paper on Intubation of the Larynx. In the after-treatment of these cases he believed it was very important only to give nourishment by enemata, and the internal use of mercury was also of much benefit. Many more of these patients would recover, he said, if more attention was given to the after-treatment.

Dr. C. E. Sajous, of Philadelphia, read a paper entitled A New Method of Intubation of the Larynx. He uses tubes having almost as large a lumen as the larynx itself, and owing to their shortness it is claimed that there is less danger of pushing down membranes. The head of the tube is so constructed that it is said to obviate any interference with deglutition, and it cannot be expelled by coughing or forced into the trachea. These tubes have been used in the cadaver, but not yet put to actual practice. In the discussion which followed, Dr. J. Solis Cohen, from Philadelphia, said he did not wish to discourage new inventions, but in his experience there were many instruments which could be used successfully upon the cadaver and yet failed upon the living subject, so he preferred to withhold his opinion concerning these tubes until further trial.

Among other papers was one by Dr. J. O. Roe, of Rochester, on Hay Fever, in which he stated that this disease always originated in the nasal passages and that local measures to correct the morbid conditions of these parts were necessary. For this purpose he was highly in favor of using chromic acid to destroy hypersensitive patches. General treatment might also be necessary. Dr. Mackenzie, of Baltimore, regarded this disease as a neurosis and favored general rather than local treatment. When practicing local measures he would not destroy the nasal tissue, but would make a stiletto incision into it. Dr. Sajous believed that local destruction of tissue only produced a temporary benefit.

A paper entitled Treatment of Atrophic Rhinitis by Applications of the Galvanic Current was presented by Dr. Delovan, of New York. He places the positive pole at the nape of the neck and the negative on the nasal mucous membrane. For an electrode he uses an ordinary piece of copper wire, bent to suit the case. The congress was invited by Dr. Lefferts, of New York, to an evening performance at the Casino and afterwards to a banquet at Delmonico's.

Dr. C. A. Powers recently read a paper on Middle Meningeal Hemorrhage before the surgical section of the Academy of Medicine. A case of this nature came under his observation at the Chambers street Hospital. The patient had been found unconscious on the sidewalk, and when admitted there was a slight hemorrhage from the right ear. Later on paralysis of the left arm and leg developed and the right pupil became dilated. The scalp over the occipital region grew ecchymosed and boggy and intracranial hemorrhage was diagnosed. Operation being decided upon, a crucial incision was made in the scalp and a fissure of the parietal bone discovered through which blood was oozing. After trephining the skull a large blood clot was seen and removed. The *dura mater* was found intact, but a large cavity remained between it and the skull, and to secure good drainage a counter-opening was made in the skull at the posterior and lower part of this cavity. A rubber drainage-tube was used and the cavity packed with iodoform gauze. Strict anticepsis was employed throughout, and the patient made a slow but good recovery. The author of the paper believed that in any such case where the symptoms point to the presence of a clot, the indications are to operate at the earliest possible period. He reported seven other cases of a like nature which were operated upon, but all but two died; yet with the rapid improvements which are being made in this department of surgery, he felt that much better results may be expected in the future. Dr. Frank Ferguson, in discussing this paper, said he had made many post-mortems where this operation seemed indicated. He had been able to locate a moderate sized clot by auscullatory percussion, and he strongly advocated this plan of treatment, as under our present management most of these patients die.

The Guild of St. John is beginning to solicit money to carry on the summer's work among the poor children of the city. This organization, among other things, provides for three weekly excursions, and last summer 18,647 sick children received the benefit of a day's sail on the salt water. Physicians accompany these excursions, who prescribe for the sick and give the parents general advice regarding the care of infants during the hot weather. The Guild also controls a seaside nursery at Staten Island, where deserving children with their mothers may be sent for a couple of weeks in the summer, and 307 patients were admitted there during last July and August.

Mr. D. O. Mills has presented the city with $50,000 to be expended in the erection of a training-school for male nurses. It is intended that the institution shall occupy the site of the old morgue, near Bellevue Hospital. The building will be five stories high, and will cover a piece of ground 75 feet on the front and rear and 80 feet deep. The top floor will be devoted to the Wood museum, and it is anticipated that its erection will take place at an early date.

Some months ago a physician of this city published a paper which he had read before one of our medical societies, in which he claimed to have cured numerous cases of epilepsy and a host of other nervous and general diseases by cutting certain of the muscles of the eye. The results claimed were very brilliant, and those who heard the paper read were rather taken by surprise, and could not combat the author's theories at that time. Soon after this the Neurological Society appointed a committee, consisting of Drs. E. C. Seguin, W. R. Birdsall and M. A. Star, to investigate into the methods practiced and the reported cases of cures. These gentlemen have now been at work for over two months, but have not yet finished their investigation, and as each is known to be thoroughly competent, and men who will not stop until they have gone to the very bottom of this affair, their report is looked for with considerable interest. There has been a good deal of excitement in ophthalmological circles and much skepticism expressed regarding this matter, but as the committee are very close-mouthed men, very little in the way of information can be ascertained from them so far. The general opinion among ophthalmologists seems to be that this physician does not cut the muscles of the eye at all, but only nicks the conjunctiva over a muscle. They say that he also corrects existing errors of refraction by proper glasses, and his reported cures are explained as the result of the moral effect of pretended muscle-cutting and the glasses. This physician has a very extensive practice, and I have been told that a large number of his patients are hypochondriacs upon whom these mental impressions are very effectual in dispelling imaginary disorders. A. F.

*New York, June, 1887.*

